# Using Andersen's Behavioral Model of Health Care Utilization to Assess Contraceptive Use among Sexually Active Perinatally HIV-Infected Adolescents in Uganda

**DOI:** 10.1155/2020/8016483

**Published:** 2020-09-28

**Authors:** Scovia N. Mbalinda, Dan K. Kaye, Mathew Nyashanu, Noah Kiwanuka

**Affiliations:** ^1^Department of Nursing, College of Health Sciences Makerere University, Kampala, Uganda; ^2^Department of Obstetrics and Gynecology, College of Health Sciences, Makerere University, Kampala, Uganda; ^3^Division of Social Work and Health-School of Social Sciences, Nottingham Trent University, UK; ^4^Department of Epidemiology and Biostatistics, School of Public Health, College of Health Sciences, Makerere University, Kampala, Uganda

## Abstract

**Background:**

Contraceptive practices of perinatally HIV-infected adolescents (PHIAs) have implications related to pregnancy prevention, risks of HIV heterosexual transmission, reinfection, and vertical transmission. The study assessed contraceptive use among sexually active PHIAs in Uganda.

**Methods:**

Mixed methods consisting of a survey and in-depth interviews were employed among 213 sexually active PHIAs who were attending antiretroviral therapy (ART) clinics. The study was guided by Andersen's Behavioral Model of Health Service Use as a theoretical framework to identify factors that influence contraceptive use. These factors include health care factors, personal characteristics, enabling factors, and needs. The outcome was contraceptive use. Multivariable logistic regression was used to establish determinants of contraceptive use. Qualitative data were analyzed by thematic analysis.

**Results:**

Most PHIAs were female (67.6%); the mean (SD) and median (IQR) age was 17.5 (±1.4) and 18 (17-19) years. The mean age of sexual debut and at marriage were 15 (±1.7) and 17 (±1.1), respectively. Condoms were the most known method of family planning (indicated by 55.4%). Only 16.9% of the participants knew about dual protection (condom use for FP as well as HIV/STI prevention). Of the PHIAs, 43.6% had ever used modern contraception and 56.9% of the females had ever been pregnant. The odds of contraceptive ever-use were significantly higher among adolescents aged 17-19 years (OR 5.1, 95% CI: 2.1-13.3) compared to those aged 10-16 years, those in school (OR 1.8, 95% CI: 1.07-3.2) compared to those out of school, and those with perceived need to use FP (OR 2.0, 95% CI: 1.1-3.9) compared to their counterparts. The odds of contraceptive used were lower among females (OR 0.13, 95% CI: 0.06-0.28) compared to males. From the in-depth interviews, the attitude of health workers, availability of health workers, having a friend using family planning, and waiting time were viewed to affect contraceptive use.

**Conclusion:**

Contraceptive use among sexually active perinatally HIV-infected adolescents was (43.6%). However, out of those who used family planning majority were using short-term methods. The unmet need for family planning was high (47%) with high reports of pregnancy (56.9%). The factors associated with contraceptive use included education, age, sex (predisposing factors), and perceived need of family planning (need factors). Other factors that could affect contraceptive use from qualitative analysis included attitude of health workers, availability of health workers, having a friend using family planning (predisposing factors), and waiting time (health system factors). HIV care for adolescents should be promoted using SRH approach. There is a need to provide training for all providers to cater for SRH services. We should continue to provide youth-responsive adolescent sexual and reproductive health services across all ART facilities and build a supportive environment and continue to integrate SRH services into HIV care.

## 1. Introduction

Globally, 1.8 million adolescents (10–19 years) were living with HIV, and 85% of them are living in sub-Saharan Africa [1]. In Uganda, we have 110,000 adolescents living with HIV [2]. Adolescents account for about 4 percent of all people living with HIV and about 11 percent of new adult HIV infections. Out of 900,000 adolescents (10-14) living with HIV, the vast majority acquired it through vertical transmission [1]. In Uganda, an estimated 110,000 adolescents were living with HIV [2]; this includes all adolescents; however, majority are perinatally infected.

PHIAs have special sexual and reproductive health needs [3]; in particular, their contraception practices have significant social consequences in HIV transmission such as the risk of heterosexual transmission, reinfection, and vertical transmission [4]. The need for adolescents' contraception is highlighted by the high rate of maternal death secondary to unsafe abortions, which account for nearly half of all maternal deaths [5]. In Uganda, adolescent pregnancy rate in the general population stands at 24% and contraceptive prevalence rate for adolescents is at 10% [6]. A study done among HIV perinatally adolescents found out that 34% were sexually active and 49.3% of PHIAs had been pregnant or made someone pregnant showing low use of contraceptives [7, 8]. There is limited data on contraceptive use among sexually active PHIAs. Also, sexual activity in HIV-positive adolescents outside the context of marriage may lead to stigma [9]. Furthermore, the nature of adolescents' sexual relationships is often sporadic and tentative combined with social taboos around becoming sexually active which presents additional barriers to seeking contraceptive advice and services [9].

Perinatally HIV-infected adolescents need special attention [3]. Their sexual and reproductive health need practices have significant social consequences concerning unwanted pregnancies and the risk of spreading HIV including superinfection and vertical transmission [4]. The standard recommendation of contraception among this population is dual protection, but it is practiced by a minority of adolescent couples [10, 11]. Dual protection among HIV-infected adolescents ranges from 16 to 18% [11, 12]. Understanding the contraceptive practices of PHIAs would help service providers to identify potential interventions for unmet needs. Since unmet needs for family planning and the HIV epidemic are driven by similar root causes, including poverty, poor access to health care, gender inequality, and social marginalization of vulnerable populations [13, 14]. This study explored contraceptive use among sexually active PHIA and factors associated with contraceptive use in Uganda.

## 2. Methods

### 2.1. Study Design and Setting

This was a mixed method study of PHIAs aged 10-19 years receiving care and treatment in 12 antiretroviral therapy (ART) clinics in three out of four regions (Eastern, Western, and Northern). The data was collected from September 1, 2013, to March 30, 2014. The mixed method included a survey on SRH experiences and 24 in-depth interviews with adolescents about their experiences of contraception services in the ART clinic. We followed the methods that have been described in the published paper in 2018 [15].

### 2.2. Participant Selection

Three regions were purposefully selected. The central region was excluded because the previous studies on PHIV adolescents were conducted in this region. In each region, four health facilities were selected. The regional referral hospitals were purposefully selected from each region because they receive referrals from the lower level facilities. To select the remaining facilities, a sampling frame comprising all health facilities in each region with 50 clients or more aged <15 years was considered [16]. Three health facilities were then randomly selected from each of the regions.

The inclusion criteria for participants were being adolescents aged 10-19 years and known HIV-positive serostatus. At each facility, the participants who met the inclusion criteria and consented to participate in the study were selected through a consecutive sampling procedure. At each site, a research assistant recruited all the participants who were available in the ART clinic until the sample size was obtained. To verify and confirm the PHIV status of the participants, medical records were checked to ascertain the HIV positive result, a positive test from parents, and the absence of information that HIV was acquired sexually or through injections. The age at which the adolescents started attending the ART clinic was also reviewed and recorded. The research assistants were trained on interview skills and ensuring privacy and confidentiality. Trained research assistants interviewed the participants in the absence of their parents or guardians.

## 3. Data Collection Methods

### 3.1. Survey on Family Planning Use among Perinatally Infected Adolescents

**Figure 1:**
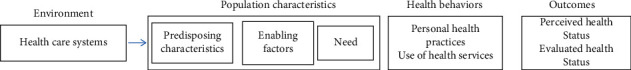
Anderson's Behavioral Model of Health Services.

An interviewer-administered questionnaire was used to collect data through face-to-face interviews. The study was guided by Andersen's Behavioral Model (ABM) of Health Service Use to guide the selection of independent variables in the present study [17, 18] (Figure 1). The model suggests environmental (i.e., external environment and health system) and person characteristics (i.e., predisposition of people to use services, factors that enable or impede this use, and a person's perception of need for care) combine to influence health behavior (i.e., personal health practices and health service use), which influences health status outcomes (i.e., perceived and evaluated health status).

**Figure 2:**
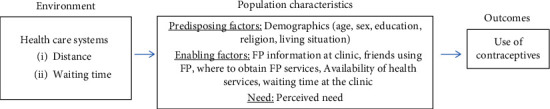
Independent variables and outcome variables mapped on to the theoretical framework: Andersen's Behavioral Model of Health Service Use. FP = family planning.

Based on the ABM and the literature reviewed, we hypothesized that the following factors would predict contraceptive use: health system factor—distance to the clinic; predisposing factors—age, education, religion, contraceptive knowledge, and attitudes toward contraceptive use; enabling factors—FP information at clinic, friends using FP, where to obtain FP services, availability of health services, and waiting time at the clinic; and need factor—perceived need of family planning, and the outcome was contraceptive use (ever used contraceptive and current use of contraceptive) (Figure 2).

### 3.2. Qualitative Data Collection

The Principal Investigator (PI) approached the adolescents in the waiting area of ART clinic to solicit interest in the study. Once an adolescent had shown interest, they were asked to remain after their ART clinic appointment for informed consent procedures and the discussion.

A total of 24 in-depth semistructured interviews (IDIs) were conducted both in English and in respective local language for adolescents who could not speak English. In order to maintain representation from each site, 2 participants (female and male) were selected by convenience sampling. Twelve in-depth interviews were also conducted with health workers in English. Health workers were purposively selected based on their role and responsibilities in ART clinic. All the identified health workers are accepted to participate in the study. The interviewees included doctors, nurse, midwife, and counselors. Interviews with health care workers were individually scheduled and were conducted within the health centre in a private room. Inclusion in the study was preceded by written informed consent for participation from key informants. All key informants were literate and conversant in English. Issues explored included sexual experiences and contraceptive use (knowledge of family planning, ever use of contraceptive methods, dual protection, access to contraceptive use, cost of contraceptive use, comfort of using contraceptives FP information at clinic, friends using availability of health services, and waiting time at the clinic).

After the opening question, discussion ensued with an aim of getting more in-depth access and availability of SRH services in Uganda. A pretested interview guide was used for both adolescents and health care workers. IDIs were conducted in private locations preferred by the interviewees within the health facility and lasted about 45-60 minutes. All interviews were audio-recorded after obtaining the participants' consent. The PI and research assistant conducted all in-depth interviews in English. The study was stopped when there were no new ideas coming up which were characterised by participants offering the same responses. Audio-recorded interviews were transcribed and translated into English by the PI as part of data familiarization and were verified by research assistants. The research assistants verified and proofread the transcribed and translated data against the recording for completeness of the transcripts and proofread the transcripts against the recorded interviews. The PI then discussed points of divergence with the research assistants for the final transcripts.

### 3.3. Data Management and Analysis

Survey data was collected from 624 perinatally HIV-infected adolescents, and out of these 213 were sexually active [7]. Data presented in this paper is a subanalysis of 213 adolescents who were sexually active. Participant's age was categorized into 10-16 years and 17-19 years. Education status was categorized into in school and out of school, and education level was categorized into no education, primary, and secondary. Occupation was grouped into three categories: students, unemployed, and volunteers (employed). Distance to the clinic was categorized into up to 5 km and more than 5 km.

The proportion of contraceptive use (those adolescents have ever used and currently using FP) was analyzed. Pearson's chi square was used to measure the association between contraceptive use and categorical and continuous variables. Stepwise logistic regression models were estimated to identify independent predictors of contraceptive use. Predictor variables with a *P* value of ≤0.2 [19] at bivariate analysis were considered for inclusion in the multivariate models. Model goodness-of-fit was assessed by likelihood ratio tests. Collinearity was assessed using a correlation matrix and cross-checked by the use of variance inflation factor which was set at 10 [20]. Variables (occupation, education status, current level of education, and highest level of education hoped to complete) were highly correlated. All the variables were dropped, and education status was left in the model. In case two variables were associated (*P* < 0.05), the variable explaining the largest variability (smaller *P* value at univariate analysis) was retained. Statistical significance was set at two-tailed *α* of 0.05, and all of the analyses were done using STATA®12.

### 3.4. Qualitative Data

Thematic analysis was conducted [21] from transcriptions of the in depth-interviews separately. The transcripts were read, and phrases of text in which there were words with similar meanings were grouped into categories. Similar categories were aggregated into themes and subthemes. The findings contain direct quotes from participants, and the narrations are reported as they were spoken by participants to maintain the meaning. A theme was identified as a consistent pattern found in the information that described. Data were analyzed and managed using NVivo version 9.

### 3.5. Ethical Review and Approval

Ethical reviews and approval were obtained from the Higher Degrees and Research Ethics Committee of the College of Health Sciences at Makerere University and the Uganda National Council for Science and Technology (REC REF 2012-085). Administrative clearance and permissions were also obtained from the management of each of the health facilities. Written informed consent was obtained from adolescents above 18 years and health providers. Adolescents below 18 years assent from the adolescents, and consent from parents or guardians was obtained. Family planning or screenings for sexually transmitted infections (STIs) were offered by referral to all participants that needed them. Participation was voluntary, and all the interviews were conducted in privacy and participant's confidentiality was maintained throughout the study by giving an identification number on the questionnaire.

## 4. Results

Generally, the mean (SD) age of the respondents was 17.5 (±1) years, and the median age was 18 years (IQR 17-19). Among them, 144 (67.4%) were females; 169 (79.3%) were aged 17-19 years; 110 (51.6%) were out of school. Regarding living status, 98 (46.0%) were living alone and were total orphans and 47 (21.6%) were married (Table 1). The mean (SD) age at first sex was 15 (±1.7) while that at marriage was 17 (±1.1) years. Twenty-four IDIs were conducted with HIV perinatally infected adolescents, 12 from females. The IDIs for service providers were 12 and 8 who were females and males, respectively.

In the study, data on knowledge on contraception was collected by asking question on whether adolescents knew about any contraceptive method. The most common known method was condom (118 (55.4%)) followed by injectable (78 (26.6%)), oral pill (78 (36.6%)), permanent methods (23 (10.8%)), intrauterine devices (IUDs) (9 (4.2%)), and traditional methods which were the least known methods compared to modern methods (Table 2). Only 36 (16.9%) had knowledge about dual protection.

Overall, 93 (43.6%) had ever used any form of modern contraceptives of which 19 (19.5%) had ever used oral pills, 60 (61.7%) had ever used condoms, and 11 (11.3%) had used injectable (Depo-Provera). Only 2 (2.1) and 5 (5.2%) had used implants and dual protection, respectively (Table 2). Out of the 144 females, 82 (56.9%) had ever been pregnant and 21 (30.4%) of the males reported that they had ever made someone pregnant. Majority of the respondents knew where to obtain family planning services (190 (89.2%)).

The adjusted odds of ever use of modern contraceptives were twice higher among adolescents aged 17-19 compared to those aged 15-16 years (AOR = 5.3, 95% CI: 2.1-13.3) (predisposing factor). Females were 87 percent less likely to have ever used contraceptives (AOR = 0.13, 95% CI: 0.06-0.28) compared to males (predisposing factor). Adolescents in school were nearly twice more likely to have ever used contraceptives (AOR = 1.8, 95% CI: 1.07-3.2) compared to those out of school (predisposing factor). Adolescents with a perceived need to use contraceptives were twice as likely to have ever used contraceptives (AOR = 2.0, 95% CI 1.07-3.9) compared to those with no perceived need to use FP (need factor) (Table 3).

### 4.1. Enabling Factors

The qualitative study mainly explored the enabling factors to access to family planning services in ART clinics. The adolescents reported that they are given the information by their health providers about family planning. However, hindrances to utilization include the attitudes of health workers, which deters adolescents from asking about contraceptives, choosing a method of their choice, or using contraceptives, as stated below.

“I need them…. but because of the rudeness of the doctors and nurses, I get scared to ask for them. One time I asked a nurse if they had any other family planning method apart from condoms, she looked at me and I never asked again.” (Femalefemale, 15-19 years).

Although it is not always the case, the provider bias often has a judgmental tone towards adolescents and young people regarding family planning usually due to their status, social norms, and culture which affects contraceptive use among young people.

In some health facilities where health providers were trained and provided SRH services, the adolescents found availability of multiple services as an enabling factor that positively influences contraceptive choice and use, as expressed below.

“There is room where we go. When you go there suffering from any sexual and transmitted diseases, they treat it. There is also family planning like condoms” (male, 15-19 years).

The adolescents also expressed long waiting time and the scarcity of health workers as hindrance to access SRH services especially family planning. Absence of the needed services often led to referral to other clinics, which often could only be accessed on another day or at another time.

“Every day, there are many clients with few health workers. This makes us spend a whole day in the clinic. When you want other services they send you to another clinic in the hospital and you cannot go to another clinic until you finish the business in the ART clinic” (male, 15-19 years).

During IDIs, adolescents expressed a need to have peer support groups because they were able to share ideas freely with their fellow adolescents. They reported that being with fellow adolescents made them feel better and they give advice to each other. Peer support groups were identified as a factor that positively influences the use of contraceptives.

“One time I found my friend with tablets in her bag and she explained these were pills to prevent pregnancy. She explained to me where she got the pills and from that day I have also started using the pills to prevent pregnancy” (female, 15-19 years).

Adolescents expressed the messages on family planning found at the clinics and on mainly targeted adults apart from condoms.

“When I hear the messages about contraceptives that are aired on the radio, I think they target adults especially women. There is no message you hear on radio targeting adolescents. At the clinic they emphasise condom use but I want to know more about other methods so that I can use it now or in the future …” (female, 15-19 years).

## 5. Discussion

Using an adapted version of Andersen's Behavioral Health Service Use model [17, 18], we found that among Ugandan sexually active perinatally HIV-infected adolescents, the factors associated with contraceptive use were education status, age, sex and perceived need of family planning. From the qualitative analysis, attitude of health workers, availability of health workers, having a friend using family planning, and waiting time affected the use of contraceptives.

Knowledge on contraceptive methods refers to whether a respondent had heard of or knows of a family planning method or methods. Contraceptive knowledge precedes contraceptive use therefore, important to know whether adolescents had knowledge on specific contraceptives. In this study, the most known method of family planning was condom and only 16.5% knew about dual protection. Sexual activity without dual protection would likely result in pregnancy, reinfection and acquiring sexually transmitted infection defeats the efforts made on HIV prevention and poor maternal outcomes. A study has shown that dual protection is lower among young people [22]. Perinatally HIV-infected adolescents should be encouraged to use dual methods at every episode of vaginal sex so as to prevent both pregnancy and reinfection and infecting others.

Education increases autonomy and decision-making skills about reproductive health choices [23]. Our study showed that adolescents in school were more likely to use contraception. A program conducted among adolescent girls providing scholastic materials improved school enrolment, reproductive health knowledge, and contraceptive use [24]. There is a need to design programs to keep perinatally HIV-infected adolescents in school so as to increase their autonomy in reproductive health decision-making. However, these interventions should be age-specific since a program in Ethiopia showed that offering scholastic materials had a much greater impact on adolescents aged 10-15 than those aged 15-17 [24]. Effective contraception promotes positive educational and economic outcomes for women and girls and is key to achieving gender equality, empowering women, and reducing poverty [25].

Peer-to-peer education programs were found to be effective in changing adolescents' knowledge and attitudes [26, 27]. Our study showed that some adolescents started using family planning because their fellow peers were using contraception. Studies have shown that adolescent reproductive health peer education programs increased reproductive health knowledge, use of contraceptives, willingness to buy contraceptives, and self-efficacy in contraceptive use [27, 28] There is need to promote peer-to-peer programs in the ART clinics looking after adolescents and also develop peer educators because studies have shown that peer educators do not only benefit others but also do benefit themselves like receiving special training in making decisions, clarifying values, and also serving as positive role models who can demonstrate the agency needed to interact with peers and navigate sexual pressures and behaviors [29], being recognized as leaders by their peers and their community, having direct involvement, a voice, and some control in programs' design and operation, learning important skills, including facilitation and communication and committing to responsible sexual behavior [29–32].

Some of the barriers for adolescents to take up SRH services more specifically to family planning are the waiting time [33, 34] and health care providers' attitude [35]. In our study, we found that waiting time affected the uptake of contraceptives as well as the attitudes of health care providers. This could be attributed to the fact that these adolescents were attending ART whose core business was HIV care but efforts should be made that adolescents set all their services at one stop centre and in limited time. This could be achieved by integrating family planning services in ART care [36], increasing the staff trained in adolescent friendly services [37], and improving staff interpersonal relationships [37]. It is fundamental to provide high-quality contraceptive care to adolescents living with HIV by providing a wide range of contraceptive methods and method choice, linking to other health services and providing the service, and considering human rights in high-quality, client-centred contraceptive care [38]. The Uganda Government has committed to collaborate with appropriate private sector bodies and institutions for the integration of MH/RH/FP and HIV&AIDS information and services and strengthen institutional capacity of public and community-based service delivery points to increase choice and quality of care at all levels (through staff recruitment, training, motivation, and equipment) [39]. It has further committed to support skill training especially through development and professionalization of midwifery [39]. This will go long in improving the attitudes and skills of health providers to provide family planning.

Health care providers play a significant role in the provision of information and service in family planning. However, the paediatric health care providers might have lacked updated family planning knowledge and furthermore lack practical experience. Thus, they might have been reluctant to put their knowledge into practice and counsel the adolescents on issues of family planning [40–44]. Therefore, continuous professional development in the area of sexual and reproductive health should be made available to health care providers caring for adolescents and young women [41]. In this study, majority of the adolescents who used family planning used short-term methods of family planning which we mainly explain as owing to misconception about the method among health care providers [43–45]. In Uganda, majority of women are offered all major modern contraceptive methods at public health facilities [6]. However, IUD is not a common choice among women, with only 2% choosing it [6]. Furthermore, in a cohort of HIV-positive perinatally infected adolescents, no one was using IUD, despite the fact that IUD is safe and does not interact with any ART treatment.

Contraceptive use among adolescents is associated with sociodemographic characteristics like age and gender [24, 46, 47]. In this study, the older adolescents (17-19 years) were more likely to use contraceptives and females were less likely to use contraceptives. Adolescents' contraceptive use may be influenced by social norms and expectations of gender roles. The belief that a girl's primary value and role in society is to bear children can impact greatly her family planning desires and decisions. Additionally, double standards related to what is socially acceptable in regard to premarital sex place pressure on boys to engage in sexual activity and girls to remain chaste [48, 49]. This can lead to girls who engage in premarital sex feeling embarrassment and shame in regard to seeking to use family planning methods.

## 6. Limitations of the Study

The study had several limitations. First, as inherent in cross-sectional study designs, associations are shown and these do not demonstrate causality. There was a potential of recall bias or underreporting of sexual behavior since this was a self-reported behavior. Participants may have forgotten or may have been embarrassed to reveal information about their sexual activity especially for the females. The males may have exaggerated their sexuality activities (20). The information adolescents provided on their contraceptive use was retrospective, which could have led to recall bias.

Also, participants may have forgotten important information on issues like condom use and number of partners. However, the research assistants were trained on interview skills and ensuring privacy and confidentiality. Additionally, in participant selection, information was not available on the mother's HIV status; this could have introduced some misclassification bias.

In conclusion, contraceptive use among sexually active perinatally HIV-infected adolescents was (43.6%). However, out of those who used family planning, majority were using short-term methods (93%). The unmet need for family planning was high (47%) with high reports of pregnancy (56.9%). The factors associated with contraceptive use included education, age, sex (predisposing factors), and perceived need of family planning (need factors). Other factors that could affect contraceptive use from qualitative analysis included attitude of health workers, availability of health workers, having a friend using family planning (predisposing factors), and waiting time (health system factors). HIV care for adolescents should be promoted using SRH approach. There is a need to provide training for all providers to cater for SRH services. We should continue to provide youth-responsive adolescent sexual and reproductive health services across all ART facilities and build a supportive environment and continue to integrate SRH services into HIV care. The paper contributes to our understanding of contraceptive use in Uganda by identifying determinants of contraceptive use among perinatally HIV-infected adolescents.

## Figures and Tables

**Table 1:** Distribution of sociodemographic characteristics of 213 HIV perinatally infected adolescent in Uganda.

Characteristics		Frequency (*n*)	Percent (%)
Gender	Males	69	32.4
	Females	144	67.6
Age groups	10–17 years	44	20.7
	17–19 years	169	79.3
Religion	Catholic	88	41.3
	Protestant	101	47.4
	Muslim	24	11.3
Education status	Out of school	110	51.6
	In school	103	48.6
Region	Western	65	30.5
	Eastern	72	33.8
	Northern	76	35.6
Distance to clinic	Within 5 km	69	32.2
	More than 5 km	145	67.8
Parenthood status (living status)^∗^	Both parents	36	16.9
	One parent (one dead)	79	37.1
	Total orphans	98	46.0
Married	Yes	47	21.6
	No	166	78.4
Age at first sex (years)		213	15.83 (1.7)
Age when got married (years)		46	17.30 (1.1)

Some responses were missing/unknown.

**Table 2:** Family planning use among sexually active HIV perinatally infected adolescents.

Characteristics		Total (*N*)	*n* (%)
Ever had about FP	Yes	213	193 (90.6).
Contraceptive use			
Yes	Yes	213	93 (43.6)
Knowledge of FP per method			
Pill	Yes	213	78 (36.6)
Injectable	Yes	213	85 (39.9)
Implants	Yes	213	17 (8.0)
IUD	Yes	213	9 (4.2)
Condoms	Yes	213	118 (55.4)
Permanent methods	Yes	213	23 (36.6)
Contraceptive method used	Pills	93	19 (20.4)
	Injections	93	11 (11.8)
	Implants	93	2 (2.1)
	Condom	93	60 (64.5)
	Dual protection	93	5 (5.4)
Knowledge about dual protection	Yes	213	36 (16.9)
Choice of FP	Own choice	93	66 (71.0)
	Relative/Guardian	93	2 (2.1)
	Health worker	93	29 (31.2)
Unmet need of family planning	Yes	213	100 (46.9)
Ever been pregnant	Yes	144	82 (56.9)
Ever made someone pregnant	Yes	69	21 (30.4)
Place to obtain FP services	Yes	213	190 (89.2)

**Table 3:** Factors associated with family planning use among sexually active HIV perinatally infected adolescent.

Variables		Adjusted OR (95% CI)	*P* value^†^
Sex	Male	1	
	Female	0.13 (0.06-0.28)	<0.001
Age	10-16		
	17-19	5.3 (2.1-13.3)	<0.001
Religion	Catholic		
	Protestant	1.1 (0.5-2.3)	0.77
	Muslim	2.4 (1.01-5.6)	0.05
Education status	Out of school	1	
	In school	1.8 (1.07-3.2)	0.02
Living situation	Both parents	1	
	One parent (one dead)	0.9 (0.4-2.4)	0.97
	Total orphan	0.53 (0.21-1.3)	0.16
Distance to ART clinic	Less than 5 km	1	
	More than 5 km	0.9 (0.4-11.8)	0.85
Perceived need for FP	No	1	
	Yes	2.0 (1.1-43.8)	0.02

## Data Availability

The datasets used and/or analyzed during the current study are not publicly available due to some privacy reason, but are available from the corresponding author (Scovia N. Mbalinda) on request on email: snmbalinda@gmail.com.
